# PTHrP increases transcriptional activity of the integrin subunit *α*_5_

**DOI:** 10.1038/sj.bjc.6603720

**Published:** 2007-04-03

**Authors:** J A Anderson, A M Grabowska, S A Watson

**Affiliations:** 1 Division of Pre-Clinical Oncology, University of Nottingham, Nottingham, UK

**Keywords:** PTHrP, integrin, metastasis, cell adhesion, transcription

## Abstract

Increasing evidence is emerging highlighting the role of parathyroid hormone-related protein (PTHrP) during metastasis by regulating cell adhesion. The current study demonstrated that modulation of PTHrP expression by PTHrP overexpression and small interfering RNA-induced silencing resulted in changes in cell adhesion and integrin expression. RNA interference of endogenous PTHrP caused a significant reduction in cell adhesion of a breast cancer cell line to collagen type I, fibronectin and laminin (*P*<0.05) and of a colon cancer cell to collagen type I and fibronectin (*P*<0.05). Overexpression of PTHrP induced a significant increase in cell adhesion of colon (*P*<0.0001) and breast (*P*<0.05) cancer cells to the same extracellular matrix proteins. These PTHrP-mediated effects were attributed to changes in integrin expression as the differences in adhesion profile correlated with the integrin expression profile. In an attempt to elucidate the mechanism whereby PTHrP regulates integrin expression, promoter activity of the integrin *α*_5_ subunit was analysed and significant increases in transcriptional activity were observed in PTHrP overexpressing cells (*P*<0.0001), which was dependent on nuclear localisation. These results indicate that modulation of cell adhesion is a normal physiological action of PTHrP, mediated by increasing integrin gene transcription.

Parathyroid hormone-related protein (PTHrP) was initially discovered as the cause of humoral hypercalcaemia of malignancy. Subsequent research has identified several normal physiological actions including growth and development, calcium homeostasis and smooth muscle relaxation (Wysolmerski and Broadus, 1994). Parathyroid hormone-related protein acts locally in an autocrine, paracrine or intracrine manner (Broadus *et al*, 1985; Burtis *et al*, 1987; Suva *et al*, 1987) with intracrine activity being the result of translocation to the nucleus, mediated by a bipartite nuclear localisation sequence (NLS) within the mid-region of the PTHrP peptide (Nguyen and Karaplis, 1998). Nuclear-localised PTHrP has been shown to be both mitogenic and antiapoptotic (Aarts *et al*, 2001; Tovar Sepulveda *et al*, 2002).

Increased expression of PTHrP has been described in a variety of tumour types, including gastrointestinal (GI) (Sidler *et al*, 1996; Alipov *et al*, 1997), but is most commonly associated with breast cancer. Furthermore, as high PTHrP expression has been identified in subsequent metastases (in particular bone), it has also been suggested that PTHrP plays a key role in tumour progression (Southby *et al*, 1990).

Ye *et al* (2001) examined the role of PTHrP in tumour growth and progression in a colon cancer cell line and demonstrated that overexpression of PTHrP in HT29 cells caused an increase in cell adhesion to the extracellular matrix (ECM) protein collagen type I. Adhesion to fibronectin and laminin was unaffected which led the authors to suggest that this selective increase in adhesion facilitated tumour cell metastasis to specific organs.

Subsequent investigations conducted by Shen and Falzon utilised cell lines derived from three different tissues (prostate, colonic and breast; PC3, LoVo and MCF-7, respectively), which showed that overexpression of PTHrP causes a significant increase in prostate and colon cancer cell adhesion to collagen type I, fibronectin and laminin and an increase in breast cancer cell adhesion to laminin only (Shen and Falzon, 2003a, 2003b, 2005; Shen *et al*, 2004). Shen and Falzon explored the mechanism by which PTHrP regulates adhesion by examining expression of the transmembrane cell adhesion receptors, integrins, which mediate cell–matrix and cell–cell adhesion (Hynes, 2002). Shen and Falzon's examination of integrin cell surface expression revealed an increase in expression of a number of integrin subunits following PTHrP overexpression and that the effect of PTHrP on integrin expression was mediated via an intracrine pathway, yet the mechanism involved was not determined.

Furthermore, it is not known whether integrin expression and adhesion are affected by endogenous PTHrP. Also, despite the suggestion that an intracrine pathway is involved, the mechanism whereby PTHrP increases integrin expression is as yet unknown.

The aims of the present study were to determine the effects of endogenous PTHrP expression on adhesion and integrin expression.

## MATERIALS AND METHODS

### Materials

The ECM proteins human fibronectin and mouse laminin-1 (derived from the EHS tumour) were purchased from BD Biosciences (Oxford, UK), whereas collagen type I (from rat tail) was obtained from Sigma Aldrich (Poole, UK). Anti-*α*_2_, -*α*_5_, -*α*_6_, -*β*_1_ and -*β*_4_ antibodies were purchased from BD Biosciences (Oxford, UK).

### Cell culture

MDAMB231 (human breast adenocarcinoma) and HT29 (human colonic adenocarcinoma) cell lines were purchased from the European Collection of Cell Cultures (Porton Down, UK) (nos. 92020424 and 91072201, respectively). MGLVA1 *ascites* is a human gastric cell line derived from a human gastric adenocarcinoma within the Division of Pre-Clinical Oncology (University of Nottingham, Nottingham, UK). All of the cell lines were cultured in RPMI 1640 medium (Sigma Aldrich) supplemented with 10% heat-inactivated fetal bovine serum and 2 mM L-glutamine (Sigma Aldrich) and grown at 37°C in a humidified 5% CO_2_ atmosphere. Semiconfluent cell monolayers were harvested using 0.025% EDTA in phosphate-buffered saline (PBS), pH 7.2 (Sigma Aldrich).

### Construction of cell lines stably transfected with PTHrP

PTHrP overexpressing cell lines were created using expression vectors containing cDNA coding for amino acids −36 to +139 of human PTHrP, which had been cloned in the sense orientation into the expression vector pcDNA3.1(+) (Invitrogen, Paisley, UK) and were a generous gift from Professor Miriam Falzon (University of Texas, TX, USA). A second construct also contained cDNA coding for amino acids −36 to +139 but was mutated in the NLS: residues 88–91 and 102–106 had been deleted using a Transformer Site-Directed Mutagenesis Kit. These two constructs along with the empty vector control were transfected into MDAMB231 and HT29 cells using Lipofectamine 2000 (Invitrogen). Briefly, for each well undergoing transfection 2 *μ*l of Lipofectamine 2000 was diluted in 50 *μ*l Opti-MEM I (Invitrogen) and incubated for 5 min at room temperature; 500 ng plasmid DNA was similarly diluted in 50 *μ*l Opti-MEM I. The DNA and Lipofectamine 2000 were combined and incubated for a further 20 min at room temperature to promote formation of transfection complexes. These complexes were then added to previously seeded cells and incubated at 37°C, 5% CO_2_. Two days following transfection, 500 *μ*g ml^−1^ G418 (Sigma Aldrich) was used to select transfected cells with individual clones grown in 96-well plates before expansion into tissue culture flasks. Clones were maintained in growth medium supplemented with G418 and tested for PTHrP expression by RT–PCR.

### RNA interference

Small interfering RNAs (siRNA) were synthesised using the *Silencer* siRNA Construction kit (Ambion, Austin, TX, USA). The target sequences are given in Table 1.

All siRNAs were transiently transfected. PTHrP siRNAs were reverse-transfected as follows: 5 × 10^4^ cells per well were seeded into 24-well plates, cultured overnight and transfected in Opti-MEM I serum-free medium using siPORT Amine (Ambion), according to the manufacturer's instructions. For each well undergoing transfection, 2 *μ*l of siPORT Amine was combined with 47 *μ*l of Opti-MEM I and incubated at room temperature for 30 min before the addition of 1 *μ*l siRNA. Unless otherwise stated, a final siRNA concentration of 800 pM was used. To allow formation of the siRNA/siPORT complexes, samples were incubated at room temperature for 15–20 min. The medium was removed from the previously seeded cells and replaced with 200 *μ*l of fresh growth medium and the transfection reagents added dropwise onto the cells.

Integrin siRNAs were forward-transfected as follows: for each well undergoing transfection, 2 *μ*l of siPORT *Neofx* was combined with 23 *μ*l of Opti-MEM I-reduced serum media and incubated at room temperature for 10 min. Small interfering RNAs were diluted to their final concentration of 5 nM (unless otherwise stated) using Opti-MEM I and then combined with the previously diluted siPORT *Neofx*. The mixture was again incubated for 10 min at room temperature to allow the formation of transfection complexes, which were then dispensed into the empty well of a 24-well plate. A 450 *μ*l portion of cell suspension (1 × 10^5^ cells ml^−1^) was then added to each well and incubated at 37°C, 5% CO_2_ to promote transfection.

### Real-time PCR

RNA was extracted using RNA-Bee (Biogenesis, Poole, UK) and reverse-transcribed from random hexamer primers (Amersham Biosciences, Little Chalfont, UK) using Superscript RT (Invitrogen). Real-time PCR was performed using the 7500 Real-time PCR System (Applied Biosystems, Warrington, UK) in conjunction with the fluorescent DNA binding dye, SYBR green. The sequences of the RT–PCR primers are given in Table 2. In addition, OAS and STAT gene expression was examined using ABI Taqman primer sets labelled with the fluorescent reporter dye FAM, which negated the need for SYBR green. An equivalent hypoxanthine guanine phosphoribosyl transferase (HPRT) set was used as a control for these primers. Relative gene expression was determined using the 2^−*ΔΔCt*^ model (Livak and Schmittgen, 2001) and HPRT used to normalise gene expression levels.

### Cell adhesion assay

Ninety-six-well tissue culture plates were precoated with 5 *μ*g ml^−1^ of either collagen type I, fibronectin or laminin in a total volume of 50 *μ*l and incubated for 1 h at room temperature. Nonspecific binding sites were blocked using 1 mg ml^−1^ bovine serum albumin (BSA, Sigma Aldrich) in PBS for 1 h at room temperature. A total of 5 × 10^4^ cells were added to each well and incubated at 37°C, 5% CO_2_ for 1 h before non-adherent cells were removed following gentle washing. The number of attached viable cells was quantified using MTT uptake as follows: cells were incubated with 1 mg ml^−1^ MTT for 4 h then excess MTT removed and the remaining formazan crystals solubilised using dimethyl sulfoxide (Sigma Aldrich). The amount of solubilised formazan product was then photometrically quantified using an MRX microplate reader at a wavelength of 550 nm and the number of viable cells used as a measure of the number of adherent cells. Results are expressed as percentage of control:

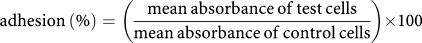


### Analysis of integrin cell surface levels by flow cytometry

A cell pellet of 2 × 10^5^ cells was washed twice with 1 ml PBA (PBS containing 5% BSA and 0.2% sodium azide) then incubated with the relevant primary antibody for 30 min at 4°C. Subsequent washes were followed by a further 30-min incubation with a FITC-conjugated rabbit anti-mouse secondary antibody (DAKO) again at 4°C. Stained cells were then washed and fixed in 1% formalin (formaldehyde in PBS). The fluorescent intensity was measured on a Beckman Coulter FACS analysis machine and analysed using WinMDI analysis software (facs.scripps.edu/software.html).

### PTHrP protein detection by Western blot

Five days following siRNA transfection, cell lysates (prepared using buffer containing 250 mM NaCl, 20 mM Tris-HCl, 0.5% Triton X-100, 3 mM EDTA, 3 mM EGTA, 1 mM PMSF, 1 mM DTT) were centrifuged at 13 000 **g** for 15 min at 4°C. The supernatant, which represents the cytoplasmic fraction, was removed and the pellet, which represents the nuclear fraction, was washed twice in lysis buffer and resuspended in RIPA buffer (137 mM NaCl, 20 mM Tris-HCl, 10% glycerol, 1% Igepal, 0.1% SDS, 0.5% sodium deoxycholate, 2 mM EDTA, 2 mM Na_3_VO_4_, 1 mM DTT, 1 *μ*M PMSF, 5 *μ*g ml^−1^ leupeptin, 20 *μ*g ml^−1^ aprotinin) used to extract the nuclear fraction. Proteins were separated on 12% NuPAGE gels (Invitrogen) before transfer to nitrocellulose membranes, which were blocked with 5% Marvel in TBS before being probed with a monoclonal anti-PTHrP (1–34) antibody at 1 *μ*g ml^−1^ (Aphton Corporation, Woodland, CA, USA) overnight at 4°C. To allow chemiluminescent visualisation using ECL reagent (Amersham), a biotin-conjugated rabbit anti-mouse secondary antibody (1:1000, DAKO) was applied to the membrane. Luminescence was visualised using GeneSnap software in conjunction with a Chemi Genius^2^ BioImaging System (Syngene) with the intensity of the bands present on the membranes being proportional to the amount of bound secondary antibody and hence amount of target protein present in the sample. Band intensities were also calculated using the Chemi Genius^2^ BioImaging System.

### Integrin reporter construct

The *α*_5_ promoter region (initially characterised by Birkenmeier *et al*, (1991)) contains consensus binding sites for a number of different elements, including Ets, Sp1, AP1 and AP2. The promoter region (from −926 to +23) was amplified using the following opposing primers, which contained restriction sites for *Kpn*I and *Bgl*II, respectively (underlined), and then cloned into a pGL3-Basic vector (Promega, Southampton, UK) using T4 ligase: CGGGTACCGTTTACACCGATTAGGAGCTGAAGGT, and GAGAGATCTTCCTAAACCTCCCAGAGGCG (Klein *et al*, 1996). Reporter constructs were transfected into cells using Lipofectamine 2000 as described above.

### Luciferase assay

Luciferase activity was measured using the Stop&Glo system (Promega, Southampton, UK). At 24 h after reporter construct transfection, cells were lysed at room temperature using 100 *μ*l of lysis buffer. The lysates were then mixed with an equal volume of Steady-Glo Assay Reagent in a solid black 96-well plate. Following a 5-min incubation at room temperature, luminescence was analysed using a MicroLumi XS Microplate Luminometer.

### Data analysis

Results from MTT adhesion assays are presented as mean±s.e.m. of 15 wells (three independent assays performed in quintuplicate) and analysed using one-way ANOVA. RT–PCR data are presented as the mean±95% confidence intervals of nine wells (three independent experiments performed in triplicate) and analysed using a Student's *t*-test. Flow cytometry results shown were analysed using one-way ANOVA and the results shown are from three independent experiments, ±s.d. Luciferase assay data were analysed using a Student's *t*-test and are presented as ±s.e.m. of nine wells (three independent assays performed in triplicate). Values of *P*<0.05 are considered significant.

## RESULTS

### Evaluation of PTHrP expression in stably transfected cell lines

To investigate the role of PTHrP in cell adhesion and integrin expression cells were transfected to overexpress endogenous PTHrP (upregulation) using constructs encoding PTHrP and PTHrP in which the NLS had been mutated through the deletion of the bipartite NLS (residues 88–91 and 102–106) by site-directed mutagenesis. These constructs were a generous gift from Professor Miriam Falzon (University of Texas). Previous studies by Falzon *et al* have shown that PC-3 cells transfected with both the wild-type and NLS-mutated PTHrP constructs secrete significantly greater amounts of PTHrP than untransfected or empty vector transfected cells (Tovar Sepulveda and Falzon, 2002). Using immunofluorescence, the same study detected nuclear and cytoplasmic staining in PC-3 cells containing the wild-type PTHrP construct with ∼60% of cells demonstrating nuclear staining; however, cells containing the NLS-mutant PTHrP exhibited strong cytoplasmic staining with nuclear staining visible in ⩽10% of cells.

**Figure 1:**
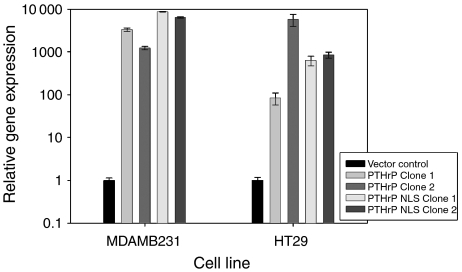
Relative PTHrP gene expression in MDAMB231 and HT29 cells stably transfected with constructs encoding for either wild-type or NLS-mutated PTHrP, expressed relative to vector control cells (*P*<0.0001).

For the present study, stable clones were established and PTHrP expression examined by RT–PCR. Parathyroid hormone-related protein gene expression in both MDAMB231 and HT29 cells transfected with the vector control was similar to that found in the corresponding wild-type cells. Cells stably transfected to overexpress wild-type and NLS-mutated PTHrP exhibited an increase in PTHrP expression between 1000- and 10 000-fold compared with vector-control cells (Figure 1, *P*<0.0001) and two individual clones for each cell line and construct were selected for further analysis.

### Knockdown of endogenous PTHrP using interfering RNA

**Figure 2:**
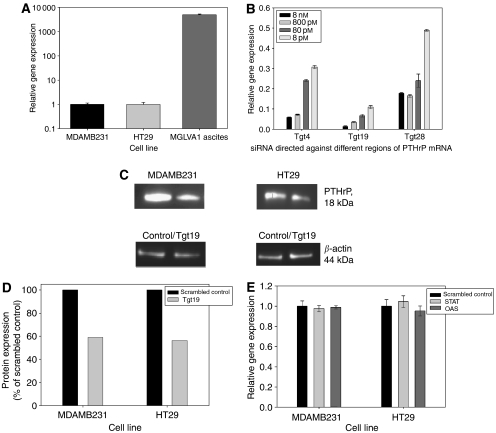
PTHrP knockdown. (**A**) Endogenous PTHrP expression in MGLVA1 ascites, MDAMB231 and HT29 cell lines, expressed relative to MDAMB231 cells. (**B**) The effect of increasing concentrations of three siRNA homologous to different regions of the PTHrP gene on gene expression in MGLVA1 ascites, 24 h after transfection and relative to GAPDH siRNA (*P*<0.0001). (**C**) PTHrP and *β*-actin protein expression in MDAMB231 and HT29 cells 5 days after transfection with 800 pM of either scrambled control or Tgt19 siRNA. (**D**) Percentage decrease in PTHrP protein expression in MDAMB231 and HT29 cells transfected with Tgt19 siRNA, as determined by densitometry analysis and expressed relative to scrambled control siRNA. (**E**) The effect of Tgt19 siRNA transfection on OAS and STAT gene expression in MDAMB231 and HT29 cells, expressed relative to scrambled control siRNA.

Initial optimisation studies of an siRNA designed to knockdown endogenous PTHrP was conducted in the gastric cancer cell line MGLVA1 ascites, which has been shown to express high levels of PTHrP (Figure 2A). To silence PTHrP expression, three siRNAs targeted against different regions of the PTHrP gene were tested at increasing concentrations for their ability to significantly reduce PTHrP gene expression (Figure 2B). All three siRNAs were able to induce a decrease in gene expression of >80% (*P*<0.0001) at the maximal concentration used; however, the most effective of the three siRNAs, Tgt19, was able to decrease gene expression by ⩾90% (*P*<0.0001) even at the lowest concentration used and was therefore used during subsequent studies at a final concentration of 800 pM.

PTHrP protein expression was also examined following siRNA transfection using Western blotting (Figure 2C). Densitometry analysis revealed a maximal decrease in protein expression of 41.0 and 43.8% in MDAMB231 and HT29 cells, respectively, 5 days after transfection with Tgt19 when compared with cells transfected with a scrambled version of the Tgt19 siRNA (Figure 2D).

Several studies have demonstrated the induction of the interferon response upon the introduction of dsRNA (Bridge *et al*, 2003; Sledz *et al*, 2003) and consequently OAS and STAT gene expression (key components of the interferon response) were examined in cells transfected with the Tgt19 siRNA. No change in expression of either gene was detected following siRNA transfection (Figure 2E).

### Manipulation of PTHrP results in significant changes in cell adhesion

**Figure 3:**
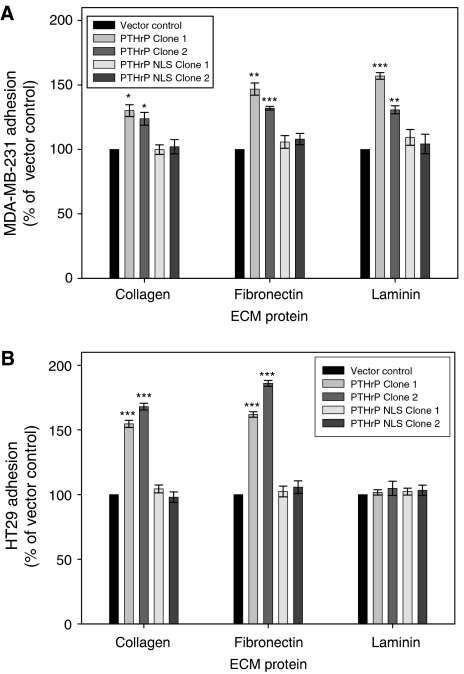
Cell adhesion of PTHrP overexpressing (**A**) MDAMB231 and (**B**) HT29 cells to the ECM proteins collagen type I, fibronectin and laminin, compared with vector control cells (^*^P<0.05, ^**^P<0.001 and ^***^P<0.0001).

Adhesion of clones overexpressing PTHrP was investigated in MDAMB231 and HT29 cells. Overexpression of wild-type PTHrP in MDAMB231 cells caused an increase in ECM adhesion: both clones demonstrated a significant increase in adhesion to collagen type I (*P*<0.05), fibronectin (*P*<0.001) and laminin (*P*<0.0001) (Figure 3A) compared with vector control cells whereas PTHrP-overexpressing HT29 cells demonstrated a significant increase in adhesion to collagen type I and fibronectin only (Figure 3B, *P*<0.0001). Despite demonstrating a significant increase in PTHrP expression, adhesion of both HT29 and MDAMB231 cells expressing NLS-mutated PTHrP was similar to the adhesion of vector control transfected cells.

**Figure 4:**
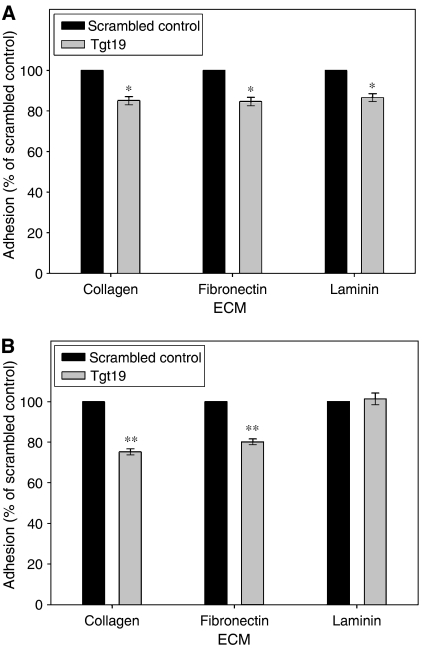
Adhesion of (**A**) MDAMB231 and (**B**) HT29 cells to collagen type I, fibronectin and laminin five days after transient transfection with either Tgt19 siRNA or scrambled control (^*^P<0.05, ^**^P<0.0001).

PTHrP was silenced in MDAMB231 (Figure 4A) and HT29 (Figure 4B) cells by siRNA and cell adhesion examined 5 days post-transfection when the protein downregulation was maximal. A small but significant reduction of ∼15% in MDAMB231 cell adhesion to collagen type I (*P*<0.05), fibronectin (*P*<0.05) and laminin (*P*<0.05) was observed. HT29 cell adhesion to laminin was unaffected by PTHrP silencing; however, there was a 25% decrease in adhesion to collagen type I (*P*<0.0001) and a 20% decrease in adhesion to fibronectin (*P*<0.0001).

### Effect of PTHrP modulation on integrin subunit expression

**Figure 5:**
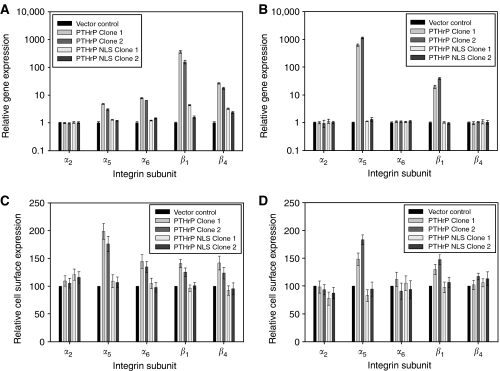
Integrin subunit gene (MDAMB231 and HT29, **A** and **B**, respectively, *P*<0.0001) and cell surface expression (MDAMB231 and HT29, **C** and **D**, respectively, *P*<0.0001) in cells expressing either wild-type or NLS-mutant PTHrP, compared with vector-control cells.

To investigate the mechanism underlying the changes in adhesion of cells over- or underexpressing PTHrP, expression of several integrin subunits was examined. Expression of wild-type PTHrP in MDAMB231 cells resulted in a significant increase in gene expression of the integrin subunits *α*_5_, *α*_6_, *β*_1_ and *β*_4_ (Figure 5A, *P*<0.0001). Expression was not significantly affected in cells expressing NLS-mutated PTHrP when compared to vector control, and no change in expression of *α*_2_ was detected in any of the clones examined.

In HT29 cells, gene expression of the subunits *α*_5_ and *β*_1_ was significantly higher in cells expressing wild-type PTHrP compared with vector control whereas expression of subunits *α*_2_, *α*_6_ and *β*_4_ was unaltered (Figure 5B, *P*<0.0001). Again, mutation of the NLS reversed the effects of PTHrP overexpression.

Integrin cell surface expression was also examined in each of the MDAMB231 and HT29 clones. In MDAMB231 cells expressing wild-type PTHrP, the change in cell surface expression corresponded with the previously observed changes in gene expression with no change in expression of *α*_2_ and a significant increase in expression of *α*_5_, *α*_6_, *β*_1_ and *β*_4_ (Figure 5C, *P*<0.0001). The integrin cell surface expression in HT29 clones also correlated with the gene expression profile with an increase in wild-type PTHrP resulting in a significant increase in expression of *α*_5_ and *β*_1_ (Figure 5D, *P*<0.0001), but no change in expression of *α*_2_, *α*_6_ or *β*_4_ in either the wild-type or NLS-mutant clones.

**Figure 6:**
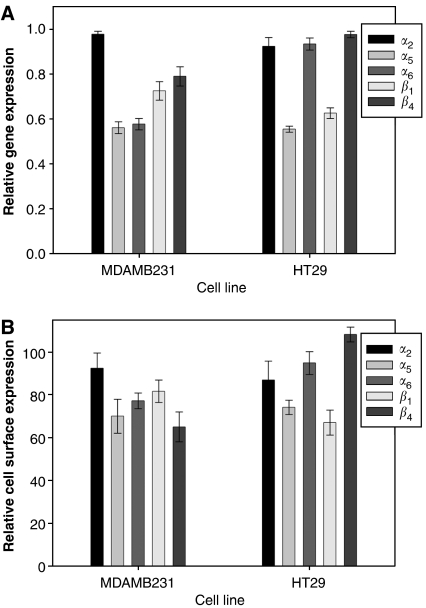
Integrin subunit gene (**A**, *P*<0.0001) and cell surface expression (**B**, *P*<0.0001) in cells transiently transfected with either scrambled control or PTHrP siRNA, results expressed relative to scrambled control.

Five days following transient transfection with the Tgt19 siRNA, there was a significant decrease in gene expression of *α*_5_ and *β*_1_ in both MDAMB231 and HT29 cells compared with scrambled control (Figure 6A, *P*<0.0001). There was also a significant decrease in expression of and *α*_6_ and *β*_4_ expression in MDAMB231 cells (*P*<0.0001).

Changes in integrin cell surface expression corresponded with the previously observed decreases in gene expression with a significant decrease in cell surface *α*_5_ and *β*_1_ expression in both MDAMB231 and HT29 cells transfected with Tgt19 and a decrease in expression of *α*_6_ and *β*_4_ in only MDAMB231 cells (Figure 6B, *P*<0.0001).

### PTHrP counteracts the effects of an integrin siRNA

**Figure 7:**
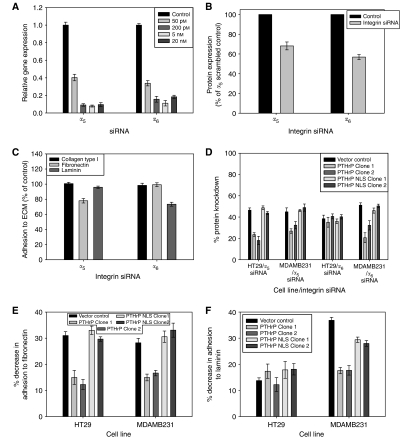
Integrin knockdown. (**A**) Effect of increasing concentrations of either *α*_5_ or *α*_6_ siRNA on respective gene expression in MDAMB231 cells, 24 h following transfection and expressed relative to *α*_5_ scrambled control siRNA (*P*<0.0001). (**B**) Integrin cell surface expression in MDAMB231 cells transiently transfected with 5 nM of *α*_5_ or *α*_6_ siRNA after 3 days, compared with *α*_5_-scrambled control siRNA (^*^P<0.001, *P*<0.0001). (**C**) Adhesion of MDAMB231 cells to ECM, 3 days after transfection with 5 nM of either *α*_5_ or *α*_6_ siRNA, compared with scrambled control siRNA (*P*<0.0001). (**D**) Effect of PTHrP overexpression on *α*_5_ cell surface expression in HT29 and MDAMB231 cell lines, 3 days following transfection with 5 nM of either *α*_5_ or *α*_6_ siRNA (*P*<0.0001). (**E**) Cell adhesion of HT29 and MDAMB231 cells to fibronectin 3 days after transient transfection with *α*_5_ siRNA, compared with scrambled control-transfected cells (*P*<0.0001). (**F**) Cell adhesion of HT29 and MDAMB231 cells to laminin following 3 days after transient transfection with *α*_6_ siRNA, compared with scrambled control-transfected cells (*P*<0.0001).

To examine further the role of integrins in PTHrP's ability to increase adhesion, the integrin subunits *α*_5_ and *α*_6_ were knocked down using siRNA. Recent work by Kim *et al* (2005) demonstrated that increasing siRNA length also increases potency and showed 27 nt to be the optimal siRNA length. When attempting to silence integrin expression, 27mer siRNAs directed towards *α*_5_ and *α*_6_ were constructed and compared with corresponding 21mer siRNAs. In each case, the longer siRNA was indeed shown to be more potent than the shorter version. For example, the 27mer *α*_5_ siRNA was able to reduce gene expression by ∼60% at the lowest concentration used (50 pM) whereas the corresponding 21mer was unable to significantly reduce gene expression (data not shown). Indeed, both the *α*_5_ and *α*_6_ 27mer siRNAs were able to reduce significantly *α*_5_ and *α*_6_ gene expression using concentrations as low as 50 pM when gene expression was examined 24 h after transfection (Figure 7A, *P*<0.0001). Similar to the gene expression results, there was a significant decrease in protein expression with both integrin siRNAs compared with scrambled control (Figure 7B, ^*^P<0.001, ^**^P<0.0001). Neither integrin siRNA induced the interferon response (data not shown).

It can be seen in Figure 7C that following transfection with the *α*_5_ siRNA there was a significant reduction in cell adhesion to fibronectin compared with the scrambled control but no decrease in cell adhesion to either laminin or collagen type I (*P*<0.0001). Similarly using the *α*_6_ siRNA, adhesion to laminin significantly decreased with transfection of the target siRNA compared with scrambled control (*P*<0.0001), whereas there was no decrease in adhesion to collagen or fibronectin. These results indicate that the downstream effects of silencing either integrin siRNA were specific to the targeted ligand only.

When protein expression was examined, overexpression of wild-type PTHrP in HT29 cells resulted in a reduction in the degree of *α*_5_ knockdown in these cells compared with vector control and NLS-deleted cells but had no effect on silencing of the *α*_6_ subunit. In HT29 vector control cells, there was a ∼50% knockdown in protein expression in *α*_5_ siRNA-transfected cells compared with scrambled control-transfected cells whereas there was only a ∼20% knockdown in wild-type expressing cells. Likewise, in MDAMB231 cells expressing wild-type PTHrP, there was a knockdown of *α*_5_ expression of only ∼30% compared with a knockdown of ∼50% in vector control cells (Figure 7D). It can also be seen in Figure 7D that the protein knockdown following *α*_6_ siRNA transfection was similar in all of the HT29 cell lines whereas in the MDAMB231 cells there was a knockdown of ∼50% in vector control cells and only a knockdown of ∼20% in wild-type overexpressing cells (*P*<0.0001).

Figure 7E and F show that when the effect of *α*_5_ and *α*_6_ siRNA transfection on cell adhesion was examined, whereas there was a significant decrease in adhesion in each of the cell lines examined, the nuclear translocation of PTHrP caused a smaller reduction in adhesion of *α*_5_-transfected HT29 cells to fibronectin (∼15% decrease compared with a 30% decrease in adhesion in vector control cells), but adhesion to laminin was similar in each of cell lines examined. Both integrin siRNA caused a significant reduction in MDAMB231 cell adhesion to fibronectin and laminin and again the presence of PTHrP within the cell nucleus appears to diminish the reduction of siRNA-induced adhesion. Both siRNA reduced adhesion to fibronectin and laminin by ∼15% in wild-type overexpressing cells, whereas adhesion to laminin was reduced by up to 40% in vector control cells (*P*<0.0001).

### PTHrP overexpression causes an increase in *α*_5_ gene transcription

Analysis of integrin expression following manipulation of PTHrP expression appeared to demonstrate that PTHrP affected integrin expression at the gene level, which was then translated to similar changes at the protein level. To investigate the mechanism involved, the promoter region of the *α*_5_ gene was cloned into a reporter construct upstream of firefly luciferase. This subunit was chosen as it had shown a link to PTHrP expression in both MDAMB231 and HT29 cells.

**Figure 8:**
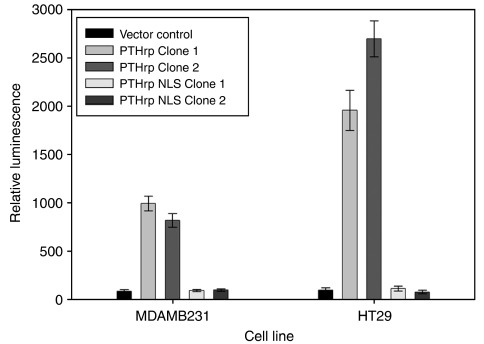
*α*_5_ promoter activity in stably transfected MDAMB231 and HT29 cells (vector, sense, NLS-mutated), as measured by luciferase assay (*P*<0.0001).

The reporter construct was transiently transfected into the PTHrP overexpressing clones and changes in luciferase activity examined. Following transient transfection with the integrin reporter construct, there was an average 10- and 25-fold increase in promoter activity in MDAMB231 and HT29 cells overexpressing wild-type PTHrP, respectively, compared with the vector control cell line (Figure 8, *P*<0.0001). Cells expressing NLS-mutant PTHrP did not demonstrate any increase in luminescence compared with vector control cells.

## DISCUSSION

The results described here show that regulation of cell–ECM adhesion via transcriptional regulation of integrin expression is a normal function of endogenous PTHrP, as well as supporting previous studies showing that overexpression of PTHrP results in upregulation of integrin expression and thereby affects cell adhesion (Ye *et al*, 2001; Shen and Falzon, 2003a, 2003b, 2005; Shen *et al*, 2004).

Cell–ECM adhesion was examined following PTHrP gene silencing and a significant reduction in adhesion to collagen type I and fibronectin observed in both a breast and colon cell line. In MDAMB231, a reduction in cell adhesion to laminin was also demonstrated.

This paralleled the data obtained using PTHrP overexpressing stable transfectants encoding for wild-type PTHrP, where overexpression in MDAMB231 and HT29 cells caused an increase in adhesion to both collagen type I and fibronectin whereas only MDAMB231 cells additionally demonstrated an increase in adhesion to laminin.

The PTHrP-induced changes in adhesion appear to be mediated via integrin subunit expression as manipulation of PTHrP expression caused parallel changes in integrin expression. When integrin subunit expression was examined following silencing of PTHrP, there was a reduction in integrin expression whereas overexpression of PTHrP resulted in a significant increase.

The observed increases in adhesion and integrin expression were not observed in cells expressing NLS-mutant PTHrP, suggesting that PTHrP's role in adhesion is mediated via a mechanism that requires its transport to the cell nucleus. The selective regulation of adhesion to ECM in the colon and breast cell lines corresponded with changes in integrin expression. In both cell lines, changes in adhesion to fibronectin and collagen were paralleled by changes in *α*_5_ and *β*_1_ expression, whereas expression of the integrin subunits *α*_6_ and *β*_4_ was only affected following PTHrP manipulation in the MDAMB231 cell line.

Following the successful design and construction of siRNA homologous to either *α*_5_ or *α*_6_, gene suppression was induced and there was a decrease in protein expression, which led to a decrease in cell adhesion. *α*_5_ forms a heterodimer with *β*_1_ to act as a receptor for fibronectin whereas *α*_6_ forms a heterodimer with either *β*_1_ or *β*_4_ and interacts with laminin (Hynes, 2002). When cell adhesion to the ECM proteins collagen type I, fibronectin and laminin was examined following gene silencing of either *α*_5_ or *α*_6_, there was a significant decrease in adhesion to only fibronectin or laminin, respectively. The downstream effects of integrin silencing were therefore specific and consistent with expectations. When the effect of PTHrP overexpression was subsequently examined, both the MDAMB231 and HT29 cells overexpressing full-length PTHrP demonstrated a smaller decrease in *α*_5_ silencing compared with both the vector control cell lines and the cell lines lacking the NLS. When *α*_6_ silencing was similarly examined, although the MDAMB231 cell lines also demonstrated a smaller decrease in silencing compared with the vector control cell lines, there was no discernable difference between the HT29 clones upon *α*_6_ silencing. As subsequent examination of protein expression and cell adhesion demonstrated a similar effect, it can be seen that PTHrP counteracts the effects of the integrin siRNA.

Consequently, these results provide further evidence of PTHrP's indirect role in cell adhesion. They also correlate with the previous findings that PTHrP's regulation of integrin expression is dependent on nuclear localisation and the observations regarding the absence of a relationship between *α*_6_ and PTHrP expression in the HT29 cell line and thereby support the hypothesis that there is tissue-specific regulation of integrin expression by PTHrP.

When Ye *et al* examined the effect of PTHrP overexpression on cell adhesion in HT29 cells, in contrast to the results described here, they showed no change in adhesion to fibronectin. Using different cell lines from those used here, Shen and Falzon showed an increase in adhesion to collagen type I, fibronectin and laminin in a colon cell line and altered adhesion to laminin only in a breast cell line following overexpression of PTHrP. Different experimental protocols were used by each group. Ye *et al* used crystal violet to quantify adhesion, Shen and Falzon used alkaline phosphatase activity, whereas MTT uptake was used in the current study. However, it is more likely that the disparities can be attributed to phenotypic differences in integrin subunit expression between cell lines and even between clones of a cell line. For example, Kiefer and Farach-Carson (2001) examined integrin cell surface expression and detected *α*_1_*β*_1_, *α*_2_*β*_1_ and *α*_3_*β*_1_ in PC-3 cells (Kiefer and Farach-Carson, 2001); this differed from results described in a similar study which found only low expression of *α*_1_*β*_1_ and *α*_3_*β*_1_ in a PC-3 clone (Kostenuik *et al*, 1997). To date, 18 *α* and eight *β* subunits have been discovered and these have been shown to form 24 different *αβ* heterodimers. Each different *α* and *β* subunit combination creates a different integrin–ligand interaction and furthermore each receptor can bind to one or more ligand, so even a small difference in integrin subunit phenotype may significantly alter a cell's adhesion profile. Shen and Falzon's examination of integrin subunit expression revealed an increase in expression of only *α*_6_ and *β*_4_ in MCF-7 cells, which corresponds with their observed increase in cell adhesion to laminin only. Similarly, PTHrP overexpressing LoVo cells demonstrated an increase in expression of *α*_2_, *α*_5_, *α*_6_, *β*_1_ and *β*_4_, and as each of these subunits can form receptors for collagen type I, fibronectin or laminin this again corresponds with the increases in cell adhesion observed. However, although the differences in adhesion profile can be attributed to differences in integrin expression it can be seen that PTHrP does not universally increase integrin subunit expression and the mechanism whereby PTHrP is able to regulate selectively integrin expression is as yet unknown.

Primary tumours appear to exhibit a partiality for one particular organ during metastasis, for example, breast cancer cells predominantly metastasise to bone (Powell *et al*, 1991) whereas GI tumours tend to establish secondary tumours in the liver (Fidler, 1990; Belluco *et al*, 2005). Parathyroid hormone-related protein expression in MDAMB231 cells demonstrated a correlation with adhesion to collagen type I, fibronectin and laminin. Bone is comprised of 95% collagen type I, with fibronectin and laminin present within the bone marrow ECM (Vituri *et al*, 2000) and laminin also a key component of the sinusoidal basement membrane (Gu *et al*, 2003), so the upregulation of integrins that bind to these ECM components may explain the fact that breast cancer cells predominately metastasise to bone (Powell *et al*, 1991). Hepatic sinusoidal endothelial cells do not possess a basement membrane but both fibronectin and collagen type I are prevalent in the space of Disse, which separates hepatocytes from sinusoids (Martinez-Hernandez and Amenta, 1995), so cells which have an increased ability to adhere to fibronectin and collagen type I (like the PTHrP expression profile of the HT29 cell line described in this report) but not laminin, may exhibit preferential metastasis to the liver rather than bone.

Previous studies have shown that intranuclear PTHrP affects proliferation (Tovar Sepulveda and Falzon, 2002), apoptosis (Aarts *et al*, 2001) and adhesion (Ye *et al*, 2001; Shen and Falzon, 2003a, 2003b, 2005; Shen *et al*, 2004). Although it has been demonstrated that PTHrP's effects on adhesion are dependent on its nuclear localisation, no mechanism has been identified to date. It has been suggested that PTHrP is a transcription factor due to the homology of PTHrP's NLS to the NLS of several established transcription factors, including c*-jun*, c*-fos* and p53 (Fiaschi-Taesch and Stewart, 2003); however, no evidence has been provided to support this hypothesis.

When the role of PTHrP in integrin transcription was examined using the integrin subunit *α*_5_, a significant increase in promoter activity was demonstrated in the cell lines overexpressing wild-type PTHrP but not in cells overexpressing NLS-mutant PTHrP. Although this result could be due to increased transfection efficiency in cells with increased integrin expression, this is unlikely as the transfection reagent used is a cationic lipid which is thought to facilitate uptake of DNA through promoting interaction of the positively charged lipid–DNA complex with the negatively charged cell membrane and uptake through endocytosis.

Not only is the present study the first to show that PTHrP plays a role in integrin transcription, but it also demonstrates that the NLS is required. This dependence on the NLS is also consistent with other results described here where the increase in adhesion and integrin expression were reversed in cells overexpressing NLS-mutated PTHrP.

During preparation of this manuscript, another study by Shen and Falzon was published examining the effect of PTHrP upregulation on *α*_6_*β*_4_ expression and Akt activation in breast cancer cells. Subsequent to demonstrating modulation of *α*_6_*β*_4_ expression at the mRNA level (consistent with the results shown here), they suggested that this indicated a ‘transcriptional and/or post-transcriptional mechanism of action for PTHrP’ and also proposed that the integrin promoter regions were necessary for PTHrP to regulate *α*_6_*β*_4_. The results described in the present study provide evidence that PTHrP does indeed act upon the integrin promoter region (in this case *α*_5_) and thereby has a transcriptional mechanism of action.

PTHrP has previously been identified as a therapeutic target with a number of studies attempting to block PTHrP to inhibit breast cancer metastasis to bone. For example, a recent study used a monoclonal antibody against PTHrP to significantly reduce osteolytic bone metastasis in a human xenograft model (Saito *et al*, 2005). The results presented here suggest that inhibiting PTHrP could help to reduce metastasis to other organs, not just bone. Furthermore, the recent success of the RNAi-based drug Cand5 in the treatment of age-related macular degeneration (Tolentino *et al*, 2004) highlights the vast potential for RNAi-based drugs in the treatment of a variety of diseases and the current study suggests that a PTHrP-specific siRNA could be similarly effective.

It is unlikely that the effects of PTHrP on integrin transcription is limited to one subunit and additional studies will be required to assess whether the effects of PTHrP on integrin transcription is limited to one subunit (*α*_5_) or whether it extends to all or a select integrin subunits. It will also be of interest to determine the precise mechanism whereby PTHrP modulates integrin expression. For instance, it is unknown whether there is a direct interaction between PTHrP and the integrin promoter sequence or whether there is upregulation of other transcription factors, which in turn act on the integrin promoter, that is does PTHrP upregulate integrin transcription in a direct or indirect manner?

In summary, this study has confirmed that PTHrP regulates cell adhesion to the ECM through modulation of integrin expression and has demonstrated for the first time that this occurs through regulation of transcriptional activity. The results described provide evidence that PTHrP is involved in integrin subunit transcription and are suggestive (albeit not conclusive) that PTHrP is a transcription factor. The precise mechanism by which PTHrP regulates integrin transcription would provide an interesting area for future research.

## Figures and Tables

**Table 1:** siRNA target sequences

**SiRNA**	**Target sequence**
PTHrP target 4	AAGGGGAAGTCCATCCAAGAT
PTHrP target 19	AAGACACCTGGGAAGAAAAAG
PTHrP target 28	AAGAAAAAACGGCGAACTCGC
PTHrP target 19 scrambled	AAGCACAAAGAGATGGAGAAC
Integrin *α*_5_	AAGAATCTCAACAACTCGCAAAGCGAC
Integrin *α*_6_	AAACATGGACCTTGATCGAAATTCCTA
Scrambled integrin *α*_5_	AACCCTCGGACCTTAAGCAGAAACAAA

PTHrp=parathyroid hormone-related protein.

**Table 2:** Oligonucleotide primer pairs used during RT–PCR

**Target gene**	**Primer sequence 5′ → 3′**
HPRT	**F** GACCAGTCAACAGGGGACAT
	**R** CGACCTTGACCATCTTTGGA
	
PTHrP	**F** GCTCGGTGGAGGGTCTCA
	**R** TTGTCATGGAGGAGCTGATGTT
	
*α*_2_	**F** AACATCCCAGACATCCCAAT
	**R** ATCATGTGATTCACCGTCAG
	
*α*_5_ (Oki *et al*, 2004)	**F** GGCAGCTATGGCGTCCCACTGTGG
	**R** CATCAGAGGTGGCTGGAGGCTT
	
*α*_6_	**F** TCAATTGCTGGAAACATGGA
	**R** GGCGGAGGTCAATTCTGTTA
	
*β*_1_ (Oki *et al*, 2004)	**F** GTGGTTGCTGGAATTGTTCTTATT
	**R** TTTTCCCTCATACTTCGGATTGAC
	
*β*_4_ (Utermark *et al*, 2003)	**F** ATAGAGTCCCAGGATGGAGGA
	**R** GTGGTGGAGATGCTGCTGTA

HPRT=hypoxanthine guanine phosphoribosyl transferase; PTHrP=parathyroid hormone-related protein; RT–PCR=reverse transcriptase–polymerase chain reaction.
